# Cyprinid herpesvirus 3: an interesting virus for applied and fundamental research

**DOI:** 10.1186/1297-9716-44-85

**Published:** 2013-09-27

**Authors:** Krzysztof Rakus, Ping Ouyang, Maxime Boutier, Maygane Ronsmans, Anca Reschner, Catherine Vancsok, Joanna Jazowiecka-Rakus, Alain Vanderplasschen

**Affiliations:** 1 Immunology-Vaccinology (B43b), Department of Infectious and Parasitic Diseases, Faculty of Veterinary Medicine, University of Liège, Liège, B-4000, Belgium

## Abstract

Cyprinid herpesvirus 3 (CyHV-3), a member of the family *Alloherpesviridae* is the causative agent of a lethal, highly contagious and notifiable disease in common and koi carp. The economic importance of common and koi carp industries together with the rapid spread of CyHV-3 worldwide, explain why this virus became soon after its isolation in the 1990s a subject of applied research. In addition to its economic importance, an increasing number of fundamental studies demonstrated that CyHV-3 is an original and interesting subject for fundamental research. In this review, we summarized recent advances in CyHV-3 research with a special interest for studies related to host-virus interactions.

## Table of contents

1. Introduction

2. Characterization of CyHV-3

2.1 General description

2.1.1 Classification

2.1.1 Morphology

2.1.1 Genome

2.1.1 Genotypes

2.1.1 Proteome

2.1 In vitro replication

2.1 Temperature restriction

2.1.1 In vitro

2.1.1 In vivo

2.1 Geographical distribution

2.1 Presence of CyHV-3 in natural environment

3. Disease

3.1 Disease characteristics

3.1.1. Clinical signs

3.1.1. Histopathology

3.1 Host range and susceptibility

3.1 Pathogenesis

3.1 Transmission

3.1 Diagnosis

3.1 Vaccination

4. Host-pathogen interactions

4.1 Genetic resistance of carp strains to CyHV-3

4.1 Immune response of carp against CyHV-3

4.2 Interferon type I response

4.2 The role of CyHV-3 IL-10 homologue

5. Conclusions

6. Abbreviations

7. Competing interests

8. Authors’ contributions

9. Acknowledgements

10. References

## 1. Introduction

The common carp (*Cyprinus carpio*) is one of the oldest cultivated fish species. In China, culture of carp dates back to at least the 5^th^ century BC, whereas in Europe, carp farming began during the Roman Empire [1]. Nowadays, common carp is one of the most economically valuable species in aquaculture: (*i*) it is one of the main cultivated fish for human consumption with a world production of 3.4 million tons per year [2]; (*ii*) it is produced and stocked into fishing areas for angling purpose; and (*iii*) its colorful, ornamental varieties (koi carp) grown for personal pleasure and competitive exhibitions represent probably the most expensive market of individual freshwater fish with some prize-winners sold for 10^4^-10^6^ US dollars [3].

Herpesviruses infect a wide range of vertebrates and invertebrates [4]. However, the host-range of individual herpesvirus species is generally restricted revealing host-virus co-evolution. In aquaculture, herpesvirus infections have been associated with mass mortality of different fish species causing severe economic losses [5-7]. In the late 1990s, a new highly contagious and virulent disease began to cause severe economic losses in both koi and common carp industries. Soon after its first known occurrences reported in Israel, USA, and Germany [8,9], the disease was described in various countries worldwide. The rapid spread of the disease was attributed to international fish trade and to koi shows around the world [10]. The causative agent of the disease was initially called koi herpesvirus (KHV) because of its morphological resemblance to viruses of the order *Herpesvirales*[9]. The virus was subsequently called carp interstitial nephritis and gill necrosis virus (CNGV) because of the associated lesions [11]. Finally, on the basis of genome homology with previously described cyprinid herpesviruses the virus was renamed cyprinid herpesvirus 3 (CyHV-3) [12].

Because of its worldwide spread and the economic losses it caused, CyHV-3 became rapidly a notifiable disease and a subject of application oriented research. However, an increasing number of recent studies have demonstrated that it is also an interesting subject for fundamental research. In this review, we summarized recent advances in CyHV-3 research with a special interest for those related to host-virus interactions.

## 2. Characterization of CyHV-3

### 2.1 General description

#### 2.1.1 Classification

**Figure 1:**
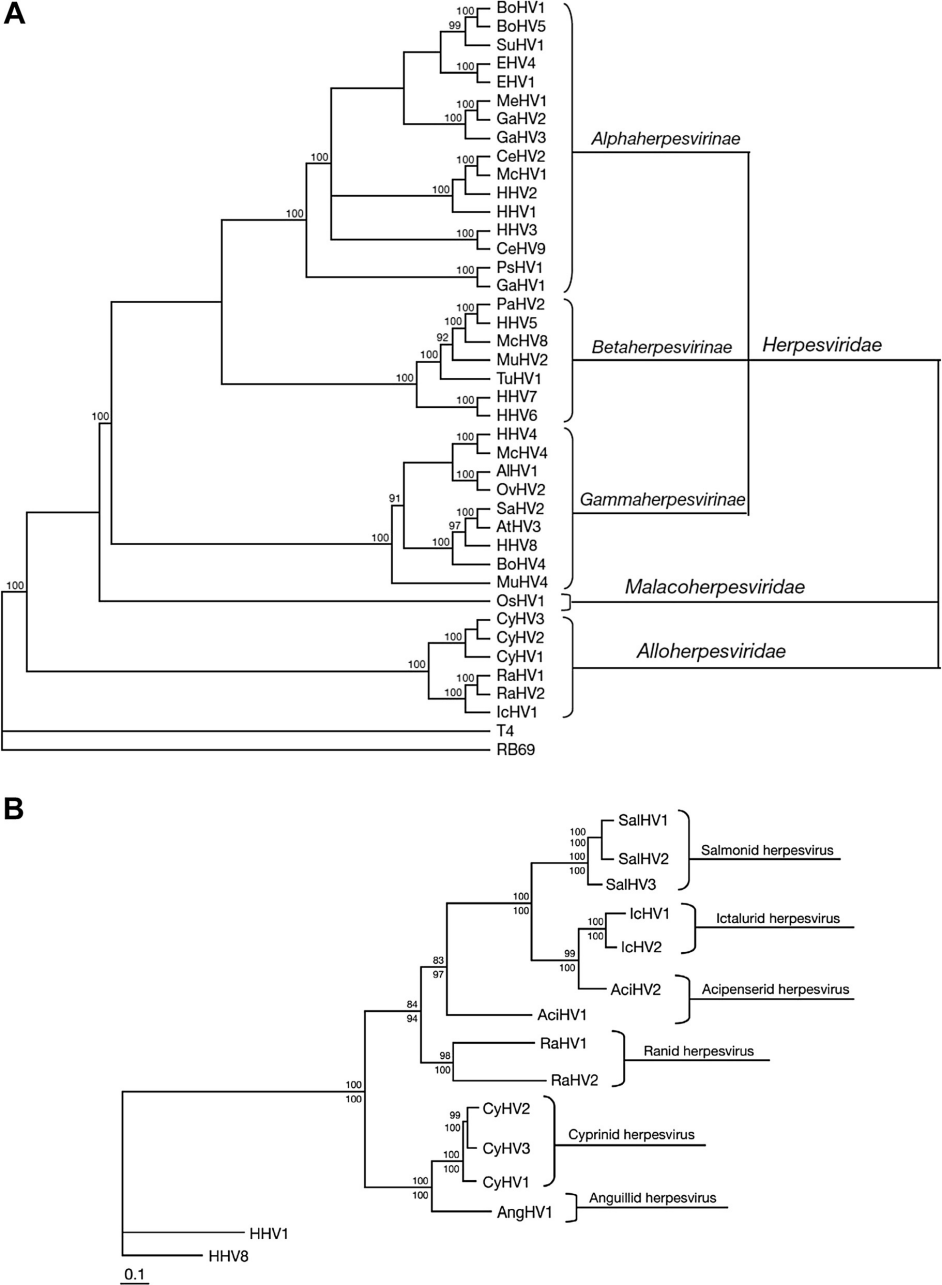
**Phylogeny of the order *Herpesvirales *and the *Alloherpesviridae *family*. *(A)** Cladogram depicting relationships among viruses in the order *Herpesvirales*, based on the conserved regions of the terminase gene. The Bayesian maximum likelihood tree was rooted using bacteriophages T4 and RB69. Numbers at each node represent the posterior probabilities (values > 90 are shown) of the Bayesian analysis. **(B)** Phylogenetic tree depicting the evolution of fish and amphibian herpesviruses, based on sequences of the DNA polymerase and terminase genes. The maximum likelihood tree was rooted with two mammalian herpesviruses (HHV-1 and HHV-8). Maximum likelihood values (> 80 are shown) and Bayesian values (> 90 are shown) are indicated above and below each node, respectively. Branch lengths are based on the number of inferred substitutions, as indicated by the scale bar. AlHV-1: alcelaphine herpesvirus 1; AtHV-3: ateline herpesvirus 3; BoHV-1, -4, -5: bovine herpesvirus 1, 4, 5; CeHV-2, -9: cercopithecine herpesvirus 2, 9; CyHV-1, -2: cyprinid herpesvirus 1, 2; EHV-1, -4: equid herpesvirus 1, 4; GaHV-1, -2, -3: gallid herpesvirus 1, 2, 3; HHV-1, -2, -3, -4, -5, -6, -7, -8: human herpesvirus 1, 2, 3, 4, 5, 6, 7, 8; IcHV-1: ictalurid herpesvirus 1; McHV-1, -4, -8: macacine herpesvirus 1, 4, 8; MeHV-1: meleagrid herpesvirus 1; MuHV-2, -4: murid herpesvirus 2, 4; OsHV-1: ostreid herpesvirus 1; OvHV-2: ovine herpesvirus 2; PaHV-1: panine herpesvirus 1; PsHV-1: psittacid herpesvirus 1; RaHV-1, -2: ranid herpesvirus 1, 2; SaHV-2: saimiriine herpesvirus 2; SuHV-1: suid herpesvirus 1; TuHV-1: tupaiid herpesvirus 1. Reproduced with permission from Waltzek et al. [14].

CyHV-3 is a member of genus *Cyprinivirus*, family *Alloherpesviridae*, order *Herpesvirales* (Figure 1A) [13]. The *Alloherpesviridae* is a newly designated family which regroups herpesviruses infecting fish and amphibians [14]. It is divided into four genera: *Cyprinivirus*, *Ictalurivirus*, *Salmonivirus*, and *Batrachovirus*[13]. The genus *Cyprinivirus* contains viruses that infect common carp (Cyprinid herpesvirus 1 and 3; CyHV-1 and CyHV-3), goldfish (Cyprinid herpesvirus 2; CyHV-2) and freshwater eel (Anguillid herpesvirus 1; AngHV-1). Phylogenetic analyses revealed that the genus *Cyprinivirus* forms a clade distinct from the three other genera listed above (Figure 1B). Viruses of the *Cyprinivirus* genus possess the largest genomes (248–295 kb) in the order *Herpesvirales*.

#### 2.1.2 Morphology

**Figure 2:**
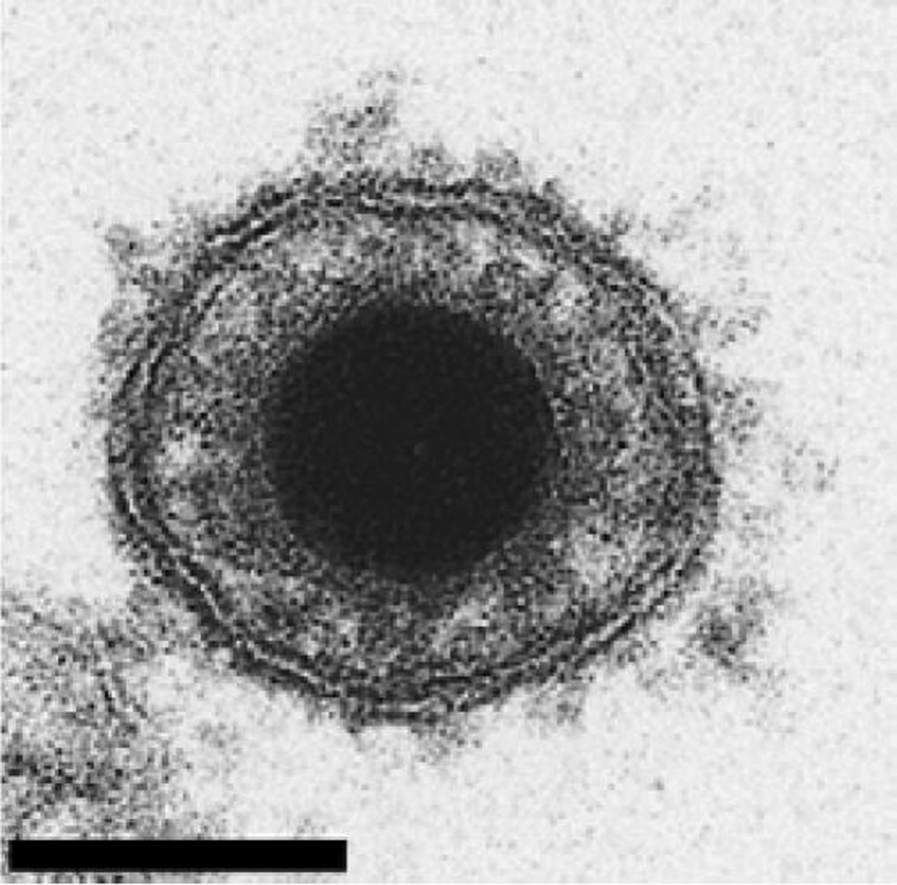
**Electron microscopy examination of CyHV-3 virion.** Bar represents 100 nm. Adapted with permission from Mettenleiter et al. [17].

Like all members of the order *Herpesvirales*, CyHV-3 virions are composed of an icosahedral capsid containing the genome, a lipid envelope bearing viral glycoproteins and an amorphous layer of proteins termed tegument, which resides between the capsid and the envelope [15]. The diameter of CyHV-3 virions is 167–200 nm according to the infected cell type (Figure 2) [15]. Morphogenesis of CyHV-3 is also characteristic of the order *Herpesvirales*, with assembly of the nucleocapsid and acquisition of the lipid envelope (derived from host cell trans-golgi membrane) that take place in the nucleus and the cytosol of the host cell, respectively [9,15,16].

#### 2.1.3 Genome

The genome of CyHV-3 is a 295 kb, linear, double stranded DNA molecule consisting of a large central portion flanked by two 22 kb repeat regions, called the left and right repeats [18]. To date, this is the largest genome among all sequenced herpesviruses. The CyHV-3 genome has been cloned as a stable and infectious bacterial artificial chromosome (BAC), which can be used to produce CyHV-3 recombinants [19].

The CyHV-3 genome is predicted to contain 155 potential protein-coding open reading frames (ORFs), among which eight (ORF1-ORF8) are duplicated in terminal repeats [13]. Nine ORFs are characterized by the presence of introns [13]. CyHV-3 genome encodes five gene families: ORF2, tumor necrosis factor receptor (TNFR), ORF22, ORF25, and RING gene families [18]. The ORF25 family consists of 6 paralogous sequences (ORF25, ORF26, ORF27, ORF65, ORF148 and ORF149) encoding potential type 1 membrane glycoproteins. Independently of the viral strain sequences, ORF26 is described as a pseudogene; while ORF27 has been characterized as pseudogene in 2 out of 3 sequenced laboratory strains [18]. All non-fragmented members of this family (ORF25, ORF65, ORF148 and ORF149) are incorporated in mature virions, presumably in the envelope [20].

Interestingly, CyHV-3 genome encodes proteins potentially involved in immune evasion mechanisms such as, for example, G-protein coupled receptor (encoded by ORF16), TNFR homologues (encoded by ORF4 and ORF12) and an interleukine-10 (IL-10) homologue (encoded by ORF134) [18].

Among the family *Alloherpesviridae*, twelve ORFs (named core ORFs) are conserved in all sequenced viruses and were presumably inherited from a common ancestor [13]. The Cypriniviruses (CyHV-1, CyHV-2 and CyHV-3) possess 120 orthologous ORFs. Twenty one ORFs are unique to CyHV-3, including ORF134 encoding an IL-10 homolog [13]. The recently described second IL-10 homolog in the family *Alloherpesviridae* encoded by AngHV-1 does not seem to be an orthologue of the CyHV-3 ORF134 [21]. CyHV-3 shares 40 orthologous ORFs with AngHV-1 although the total number of ORFs shared by all CyHVs with AngHV-1 is estimated to be 55 [13]. This supports the phylogenetic conclusion that among the genus *Cyprinivirus*, CyHVs are more closely related to each other than to other members of the family *Alloherpesviridae*[14]. Interestingly, CyHV-3 also encodes genes with closest relatives in viral families such as *Poxviridae* and *Iridoviridae*[18,22].

#### 2.1.4 Genotypes

Whole genome analysis of three CyHV-3 strains isolated in Israel (CyHV-3 I), Japan (CyHV-3 J) and United States (CyHV-3 U) revealed high sequence identity between the strains [18]. The relationships between these strains revealed that U and I strains are more closely related to each other and form one lineage (U/I), whereas J strain is more distinct and forms a second lineage (J) [18]. The existence of genetic differences between European lineage (including U and I genotypes) and Asian lineage (including J genotype) were later confirmed and suggests independent CyHV-3 introductions in various geographical locations [23,24]. Furthermore, Kurita et al. demonstrated that the Asian lineage contains only two variants (A1 and A2) while the European lineage has seven variants (E1–E7) [24]. Recently, a new intermediate genetic lineage of CyHV-3 including isolates from Indonesia has been suggested [25]. This hypothesis was later supported by analyses of multi-locus variable number of tandem repeats (VNTR). These analyses also suggested that genetically distinct viral strains can coexist in a same location following various introduction events [26]. Although previous study described presence of both CyHV-3 lineages in Europe [23], an European genotype of CyHV-3 has only been revealed recently in East and Southeast Asia [27]. Recently, Han et al. described polymorphism in DNA sequences encoding three envelope glycoprotein genes (ORF25, ORF65, and ORF116) among CyHV-3 strains from different geographical origins [28].

#### 2.1.5 Proteome

**Figure 3:**
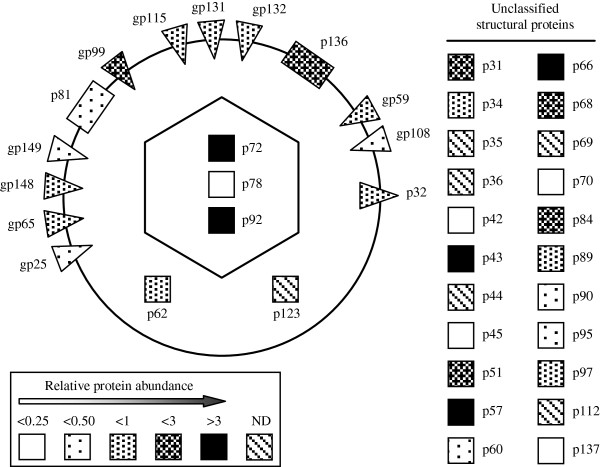
**Schematic representation of CyHV-3 virion proteome.** The viral composition of the envelope (circle), capsid (hexagon) and tegument is indicated. Membrane proteins of type 1, 2 and 3 are represented by triangles pointed inside, triangles pointed outside and rectangles, respectively. Other proteins are shown as squares. The different fillings indicate the relative abundance of proteins based on their emPAI (< 0.25, < 0.50, < 1, < 3 and > 3). p: protein, gp: glycoprotein, ND: no data. Reproduced with permission from Michel et al. [20].

Different groups used mass spectrometry to identify CyHV-3 proteins and to study their interactions with cellular and viral proteins. The structural proteome of CyHV-3 was recently characterized by using liquid chromatography tandem mass spectrometry [20]. A total of 40 structural proteins, comprising 3 capsid, 13 envelope, 2 tegument, and 22 unclassified proteins, were described (Figure 3). The genome of CyHV-3 possesses 30 potential transmembrane-coding ORFs [18]. With the exception of ORF81, which encodes a type 3 membrane protein expressed on the CyHV-3 envelope, no CyHV-3 structural proteins have been studied [20,29]. ORF81 is thought to be one of the most immunogenic (major) membrane proteins of CyHV-3 [29]. Recently, Gotesman et al. using anti-CyHV-3 antibody-based purification coupled with mass spectrometry, identified 78 host proteins and five potential immunogenic viral proteins [30]. In another study, concentrated supernatant was produced from CyHV-3 infected CCB cultures and analyzed by 2D-LC MS/MS in order to identify CyHV-3 secretome. Five viral and 46 cellular proteins were detected [31]. CyHV-3 ORF12 and ORF134 encoding respectively a soluble TNFR homologue and an IL-10 homologue, were among the most abundant secreted viral proteins [31].

### 2.2 In vitro replication

CyHV-3 is widely cultivated in cell lines derived from common carp brain (CCB), gills (CCG) and fin (CaF-2) [32,33]. Permissive cell lines have also been derived from koi fin: KF-1 [9], KFC [11], KCF-1 [34], NGF-2 and NGF-3 [16]. Non-carp cell lines, such as silver carp fin (Tol/FL) and goldfish fin (Au) were also described as permissive to CyHV-3 [35]. Oh et al. reported the expression of cytopathic effect (CPE) in cell line from fathead minnow (FHM) after inoculation with CyHV-3 [36], but this observation was not confirmed by other studies [9,35].

In vitro study showed that all annotated CyHV-3 ORFs are transcribed during CyHV-3 replication [37]. Transcription of CyHV-3 genes starts as early as 1 h post-infection and viral DNA synthesis initiates as early as 4–8 h post-infection [37]. Similar to all other herpesviruses, most of CyHV-3 ORF transcripts can be classified into three temporal kinetic classes: immediate early (IE; *n* = 15 ORFs), early (E; *n* = 111 ORFs) and late (L; *n* = 22 ORFs). Seven ORFs are unclassified [37]. Fuchs et al. demonstrated that CyHV-3 ORFs that encode for three enzymes implicated in nucleotide metabolisms: thymidine kinase (ORF55), dUTPase (ORF123) and ribonucleotide reductase (ORF141) are nonessential for virus replication in vitro [38].

### 2.3 Temperature restriction

Water temperature is one of the major environmental factors that influences the onset and severity of viral infection in fish [39]. This statement certainly applies to CyHV-3 as temperature was shown to affect drastically both viral replication in vitro and CyHV-3 disease in vivo.

#### 2.3.1 In vitro

CyHV-3 replication in cell culture is restricted by temperature. Optimal viral growth in KF-1 cell line was observed at temperatures between 15 °C and 25 °C. Virus propagation and virus gene transcription are gradually turned off when cells are moved from permissive temperature to the non-permissive temperature of 30 °C [40,41]. However, infected cells maintained for 30 days at 30 °C preserve infectious virus, as demonstrated by viral replication when the cells are returned to permissive temperatures [40].

#### 2.3.2 In vivo

CyHV-3 disease occurs naturally when water temperature is between 18 °C and 28 °C. Several studies demonstrated that transfer of recently infected fish (between 1 and 5 days post-infection (dpi)) to non-permissive low (≤ 13 °C) or high temperatures (> 30 °C) significantly reduces the mortality [11,42-44]. Water temperature was also shown to affect the onset of mortality: the first mortalities occurred between 5–8 and 14–21 dpi when the fish were kept between 23-28 °C and 16-18 °C, respectively [42,45].

### 2.4 Geographical distribution

CyHV-3 was first isolated from infected koi originating from Israel and USA in 2000 [9]. Soon after, outbreaks of CyHV-3 occurred in many countries in Europe, Asia and Africa [10,22]. Currently, only South America, Australia and northern Africa seem to be free of CyHV-3. The global and rapid spread of the virus is thought to be mainly due to the international trading of common and koi carp, but also to koi shows.

### 2.5 Presence of CyHV-3 in natural environment

In addition to its economic impact on common and koi carp industries, CyHV-3 has also a negative environmental impact by affecting wild populations of carp. In 2003, the first outbreak of CyHV-3 disease among wild carp occurred in the Yoshi river in Japan [46]. The virus then spread among several freshwater systems and caused mass mortalities in wild carp populations. In Lake Biwa, about 70% of carp population (more than 100 000 fish) died due to CyHV-3 infection in 2004 [46]. Mass mortalities of wild carp have been also described in angling waters in UK in 2003 [47], in New York and South Carolina, USA in 2004 [48,49] and in Kawartha Lakes region, Ontario, Canada in 2007 [50]. The monitoring of the distribution of CyHV-3 in rivers and lakes in Japan demonstrated that it can persist in the wild carp populations and can be subsequently transmitted to naïve fish [46,51,52]. Studies performed in habitats with CyHV-3 history suggested that sediments [53] and aquatic invertebrates feeding by water filtration could represent potential reservoirs of CyHV-3 [54]. Moreover the viral DNA could be detected in water not only during the outbreak of the disease but also for at least 3 months after the end of mass mortality [51]. However, it has to be noted that these studies relied on the detection of viral genome and not CyHV-3 infectivity. Consequently further studies are required to determine whether these potential reservoirs of infectious virus could play a role in CyHV-3 epidemiology.

## 3. Disease

### 3.1 Disease characteristics

CyHV-3 disease is seasonal, occurring when water temperature is between 18 °C and 28 °C. It is restricted to common and koi carp and their hybrids with other species [55]. It is highly contagious and extremely virulent with mortality rate that can reach 80 to 100%. Fish infected with CyHV-3 by immersion, injection or oral route and kept at 23-28 °C die between 5 and 22 dpi with a peak of mortality between 8 and 12 dpi [9,56,57]. Gilad et al. suggested that loss of osmoregulation of the gills, gut and kidney contributes to mortality during acute infection with CyHV-3 [42]. Furthermore, CyHV-3 infected fish are more susceptible to secondary infections by bacterial, parasitic or fungal pathogens which may cause further mortality within the population.

#### 3.1.1. Clinical signs

**Figure 4:**
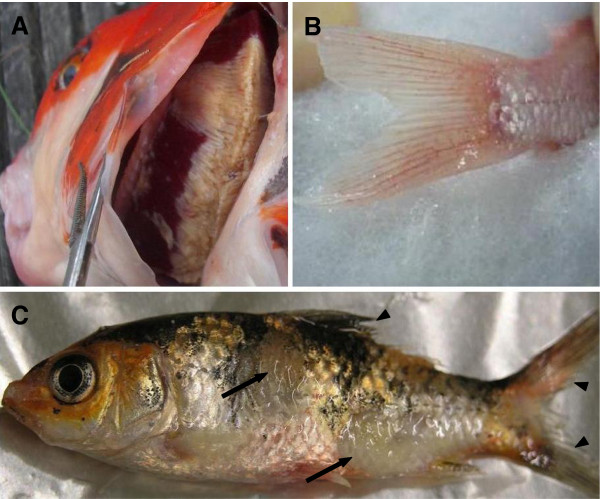
**Some of the clinical signs observed during CyHV-3 infection. (A)** Severe gill necrosis. **(B)** Hyperemia at the base of the caudal fin. **(C)** Herpetic skin lesions on the body (arrows) and fin erosion (arrowheads). Reproduced with permission from Michel et al. [22].

The first clinical signs appear at 2–3 dpi. Fish become lethargic, lie at the bottom of the tank with the dorsal fin folded and exhibit loss of appetite. In ponds, infected fish are usually gathering close to the water inlet or sides of the pond and gasp at the surface of water. Gill necrosis coupled with extensive discoloration and increased mucus secretion appear as early as 3 dpi. Depending on the stage of the infection, the skin exhibits different clinical signs, such as hyperemia, particularly at the base of the fins and on the abdomen; pale, irregular patches on the skin associated with mucus hypersecretion at the beginning of infection; peeling away of dead epithelium and lack of mucus cover in the later stage of infection; appearance of epidermis surface with a sandpaper-like texture; and herpetic lesions (Figure 4). In addition, fin erosion and bilateral enophthalmia (sunken eyes) are observed in the later stages of infection. Some fish show neurologic signs in the final stage of disease, when they become disoriented and lose equilibrium [9,10,58].

#### 3.1.2. Histopathology

The most important histopathological changes are observed in the gills. They involve erosion of primary lamellae, fusion of secondary lamellae and adhesion of gill filaments [58,59]. Gills also exhibit hyperplasia, hypertrophy and/or nuclear degeneration of branchial epithelium and congestion of the blood vessels in the gill arch [15,59]. Severe inflammation and gill necrosis resulting in the complete loss of lamellae can also be observed [31,59]. In the kidney, the hematopoietic cells are the most affected ones [15]. However, a weak peritubular inflammatory infiltrate is evident in kidney as early as 2 dpi and increases with time. It is accompanied by blood vessel congestion and degeneration of the tubular epithelium in many nephrons [59]. In the spleen and liver, the most obviously infected cells are splenocytes and hepatocytes, respectively [15]. In the liver, mild inflammatory infiltrates are observed mainly in the parenchyma [59]. In the brain, focal meningeal and parameningeal inflammation is observed [59]. Analysis of brain from fish that showed clear neurologic signs revealed congestion of capillaries and small veins associated with edematous dissociation of nerve fibers in the valvula cerebelli and medulla oblongata [15]. In the skin, the number of goblet cells is reduced by 50% in infected fish. Furthermore, the goblet cells appeared mostly slim and slender which suggests that mucus was released and had not been replenished. In addition, erosion of skin epidermis is frequently observed [60].

### 3.2 Host range and susceptibility

CyHV-3 causes a symptomatic disease only in common and koi carp. Hybrids of koi × goldfish and koi × crucian carp are also affected by CyHV-3 disease, with mortality rate of 35% and 91%, respectively [55]. Common carp × goldfish hybrids have also been reported to show some susceptibility to CyHV-3 infection; however, the mortality rate observed was rather limited (5%) [61]. PCR detection of CyHV-3 performed on cyprinid and non-cyprinid fish species, but also on freshwater mussels and crustaceans, suggested that these species could act as reservoirs of the virus (Table 1) [54,62-67]. Cohabitation experiments suggest that some of these fish species (goldfish, tench, vimba, common bream, common roach, European perch, ruffe, gudgeon, rudd, northern pike, silver carp and grass carp) can carry CyHV-3 asymptomatically and transmit it to naïve carp [64,65,68-70]. Consistent with this observation, in vitro studies showed that CyHV-3 can replicate and cause CPE in cell cultures derived not only from common and koi carp but also from silver carp and goldfish [35]. Recent studies provided increasing evidence that CyHV-3 can infect goldfish asymptomatically [68,69]. Finally, the World Organisation for Animal Health (OIE) listed four CyHV-3 susceptible species (*Cyprinus carpio* and its hybrids, goldfish*,* Russian sturgeon and Atlantic sturgeon) and two potential susceptible species (grass carp and ide) [71].

**Table 1:** Organisms tested for CyHV-3 infection.

**Common name (species)**	**Detection of CyHV-3**			**Detection of CyHV-3 genome in naïve carp after cohabitation**
	**DNA**	**Transcript**	**Antigen**	
**Vertebrates**				
*Cyprinidae*				
Goldfish (*Carassius auratus*)	Yes [62,68-70]	Yes [68]	Yes [69]	Yes [68-70]
Ide (*Leuciscus idus*)	Yes [62,63]	NT	NT	NT
Grass carp (*Ctenopharyngodon idella*)	Yes [62,64,70]	NT	NT	Yes [64,70]
Silver carp (*Hypophthalmichthys molitrix*)	Yes [64,70]	NT	NT	Yes [64,70]
Prussian carp (*Carassius gibelio*)	Yes [64,70]/ No [65]	NT	NT	Yes [70]/No [65]
Crucian carp (*Carassius carassius*)	Yes [64]	NT	NT	NT
Tench (*Tinca tinca*)	Yes [64,65,70]	NT	NT	Yes [64,65,70]
Vimba (*Vimba vimba*)	Yes [63,64]	NT	NT	Yes [64]
Common bream (*Abramis brama*)	Yes [64,65]	NT	NT	Yes [64]
Common roach (*Rutilus rutilus*)	Yes [64,65]	NT	NT	Yes [64]/No [65]
Common dace (*Leuciscus leuciscus*)	Yes [64,65]	NT	NT	No [65]
Gudgeon (*Gobio gobio*)	Yes [64,65]	NT	NT	Yes [65]
Rudd (*Scardinius erythrophthalmus*)	Yes [65]	NT	NT	Yes [65]
European chub (*Squalius cephalus*)	Yes [64]/No [65]	NT	NT	NT
Common barbel (*Barbus barbus*)	Yes [64]	NT	NT	NT
Belica (*Leucaspius delineatus*)	Yes [64]	NT	NT	NT
Common nase (*Chondrostoma nasus*)	Yes [64]	NT	NT	NT
*Acipenseridae*				
Russian sturgeon (*Acipenser gueldenstaedtii*)	Yes [66]	NT	NT	NT
Atlantic sturgeon (*Acipenser oxyrhynchus*)	Yes [66]	NT	NT	NT
*Cobitidae*				
Spined loach (*Cobitis taenia*)	Yes [64]	NT	NT	NT
*Cottidae*				
European bullhead (*Cottus gobio*)	Yes [64]	NT	NT	NT
*Esocidae*				
Northern pike (*Esox lucius*)	Yes [64,65]	NT	NT	Yes [65]
*Gasterosteidae*				
Three-spined stickleback (*Gasterosteus aculeatus*)	Yes [65]	NT	NT	No [65]
*Ictaluridae*				
Brown bullhead (*Ameiurus nebulosus*)	Yes [65]	NT	NT	No [65]
*Loricariidae*				
Ornamental catfish (*Ancistrus sp.*)	Yes [62]	NT	NT	NT
*Percidae*				
European perch (*Perca fluviatilis*)	Yes [64,65]	NT	NT	Yes [64]/No [65]
Ruffe (*Gymnocephalus cernua*)	Yes [64]/No [65]	NT	NT	Yes [64,65]
**Invertebrates**				
Swan mussels (*Anodonta cygnea*)	Yes [54]	NT	NT	NT
Scud (*Gammarus pulex*)	Yes [54]	NT	NT	NT

NT- not tested.

Carp of all ages, from juveniles upwards, are affected by CyHV-3, but younger fish (1–3 months, 2.5-6 g) seem to be more susceptible to infection than mature fish (1 year, ≈ 230 g) [58]. Ito et al. suggested that carp larvae are not susceptible to CyHV-3 since larvae (3 days post-hatching) infected with virus showed no mortality whereas most of the carp juveniles (>13 days post-hatching) died after infection [72]. However, recent study using CyHV-3 recombinant strain expressing luciferase (LUC) as a reporter gene, demonstrates that carp larvae are sensitive and permissive to CyHV-3 infection immediately after hatching and that their sensitivity increases with the developmental stages [73].

### 3.3 Pathogenesis

**Figure 5:**
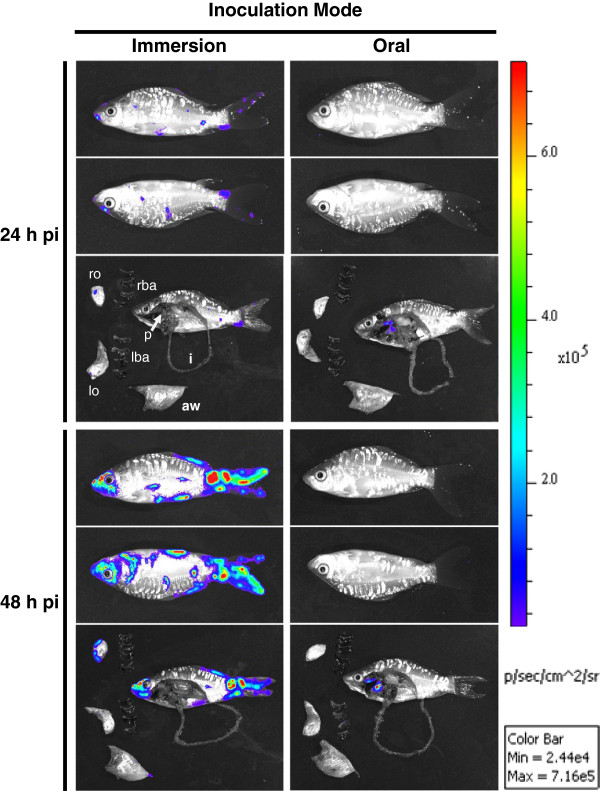
**The portal of entry of CyHV-3 in carp analysed by bioluminescence imaging.** Two groups of fish (mean weight 10 g) were infected with a recombinant CyHV-3 strain expressing luciferase as a reporter gene either by bathing them in water containing the virus (Immersion, left column) or by feeding them with food pellets contaminated with the virus (Oral, right column). At the indicated time post-infection, six fish per group were analysed by bioluminescence IVIS. Each fish was analysed lying on its right and its left side. To analyze internal signals, fish were euthanized and dissected immediately after in vivo bioluminescence imaging. Dissected fish and isolated organs were analysed for ex vivo bioluminescence. The analysis of one fish is presented for each time point and inoculation mode. Pictures collected over the course of this experiment are presented with a standardized minimum and maximum threshold value for photon flux. rba, right branchial arches; lba, left branchial arches; ro, right operculum; lo, left operculum; p, pharynx; aw, abdominal wall; i, intestine. Reproduced with permission from Fournier et al. [57].

**Figure 6:**
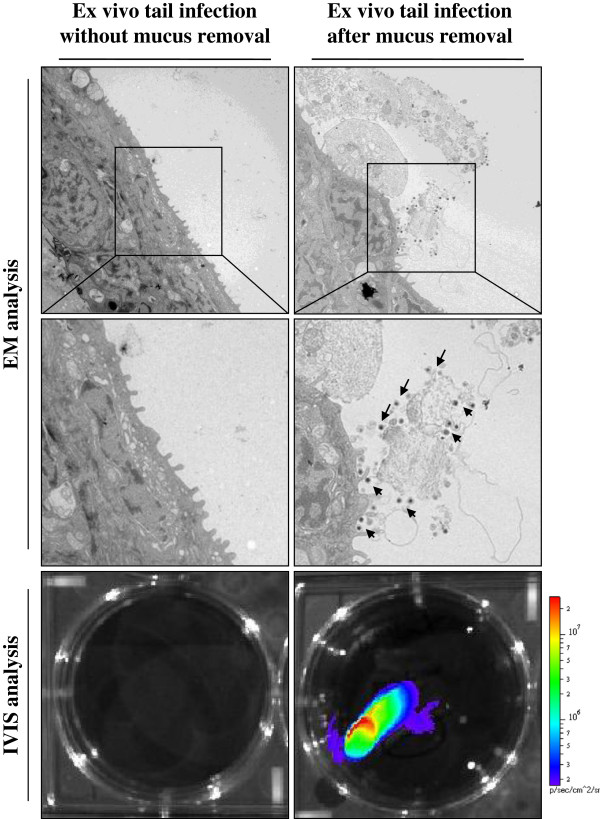
**Effect of skin mucus removal on CyHV-3 binding to carp epidermal cells.** Tail fin ventral lobes of carp were mock-treated or treated by rubbing with a soft tissue paper to remove epidermal mucus. Immediately after skin treatment, tail fin explants were harvested and inoculated ex vivo with a CyHV-3 recombinant strain expressing luciferase as a reporter gene (10^6^ PFU/mL of culture medium for 2 h). At the end of the 2 h inoculation period, a fragment of the fin was collected and processed for electron microscopy examination (EM analysis). The arrows indicate CyHV-3 particles bound to cells or cell debris. Twenty-four hours post-inoculation, duplicate tail explant cultures were analyzed by bioluminescence imaging (lower panels). Reproduced with permission from Raj et al. [75].

In early reports, it has been suggested that CyHV-3 may enter the host through infection of the gills based on detection of viral particles and viral genome in this organ as early as 1–2 dpi [42,59]. However, more recent studies using in vivo bioluminescent imaging system demonstrated that according to epidemiological conditions CyHV-3 can enter carp either by skin (immersion in infectious water) or pharyngeal periodontal mucosa infection (ingestion of infectious materials) (Figure 5) [57,74]. The epidermis of teleost fish is a living stratified squamous epithelium that is capable of mitotic division at all levels (even the outermost squamous layer). The scales are dermal structures and consequently are covered by the epidermis [74]. Removal of skin mucus and epidermal lesions facilitates the entry of virus into the host (Figure 6) [75]. After initial replication in the epidermis [74] the virus is spreading rapidly in infected fish as indicated by detection of CyHV-3 DNA in almost all internal tissues as early as 24 h post-infection [42]. The tropism of CyHV-3 for white blood cells most probably explains such a rapid spread of the virus within the body [76]. Virus replication in organs such as the gills, skin and gut represents source of viral excretion into the water. Recently, pharyngeal periodontal mucosa has been shown to be the portal of entry of CyHV-3 after infection by the oral route using food pellets contaminated with the virus [57]. This model of inoculation led to the spreading of the infection to the various organs tested as well as resulted in clinical signs and mortality rate comparable to the infection by immersion [57].

All members of the family *Herpesviridae* exhibit 2 distinct phases in their biological cycle: lytic replication and latency. While lytic replication is associated with production of viral particles, latency consists in the maintenance of the viral genome as a nonintegrated episome and the expression of very few viral genes and microRNAs. Upon reactivation, latency is replaced by lytic replication. Even if latency has not been demonstrated conclusively in members of the *Alloherpesviridae* family, an increasing number of evidences support the existence of latent phase. These evidences related to CyHV-3 can be summarized as follows. (*i*) CyHV-3 DNA has been detected in the brain of fish that survived primary infection and showing no clinical signs at 64 dpi [42], and even 1 year post-infection [77]. (*ii*) CyHV-3 persisted in the wild population of common carp for at least 2 years after initial outbreak [46]. (*iii*) Finally, St-Hilaire et al. described, in fish that survived the primary infection, the induction of CyHV-3 reactivation by temperature stress several months after the initial exposure to the virus [43]. Increased level of viral DNA in gills without the appearance of disease symptoms has been detected after stress induced by netting fish that survived the primary infection and were kept at 20 °C for 81 dpi [78]. Recent studies demonstrated that virus may become latent in white blood cells and other tissues, remains at very low copy numbers and can be reactivated by temperature stress [76,79,80]. To date, the temperature-dependent reactivation of the disease which resulted in mortality of naïve cohabitant fish has been described after transferring the fish maintained at a low temperature to the higher, permissive temperature [43,79]. These observations suggest that the temperature of the water could regulate the switch between latency and lytic replication and vice versa allowing the virus to persist in the host population throughout the seasons even when the temperature is non-permissive.

### 3.4 Transmission

To date, no evidence of CyHV-3 vertical transmission has been reported. Horizontal transmission of CyHV-3 occurs either by direct transmission (fish to fish) or vector based transmissions. Direct transmission can be by skin to skin contact of infected carp or cyprinid and non-cyprinid fish species that can carry CyHV-3 asymptomatically [64,68] against naïve carp; or by cannibalistic and necrophagous behaviors of the carp [22,57]. Several potential vectors could be involved in the vector based transmission of CyHV-3. Such vectors include fish droppings [81], plankton [82], aquatic invertebrates feeding by water filtration [54], piscivorous birds which could transfer the disease by moving sick fish from one pond to another [83], and finally the water being the major abiotic vector. Secretion of viral particles into the water either through shedding or together with sloughed epithelial cells has been documented [59]. Furthermore, the infectivity of CyHV-3 in water was shown to be conserved for at least 4 h [58], even if longer period could be observed depending on water composition (chemical and microbial) [84]. For example, the infectivity of CyHV-3 was drastically reduced after 3 days in environmental water, although it remained quite stable for more than 7 days in sterilized water [84].

### 3.5 Diagnosis

Various CyHV-3 diagnostic methods have been developed. They are based on the detection of infectious particles, viral DNA, transcripts, or antigens. Virus isolation from infected fish tissues in cell culture was the first method developed [9,11]. Although cell culture isolation is not as sensitive as PCR-based methods, it is the only technique able to detect infectious particles. Recently, Dong et al. isolated for the first time CyHV-3 virus from diseased koi in mainland China using a newly developed cell line from caudal fin of koi [34]. A complete set of molecular techniques for detection of viral DNA fragments has been developed, such as DNA hybridization, PCR, nested PCR, one-tube semi-nested PCR, semi-quantitative PCR, real-time TaqMan PCR, and loop-mediated isothermal amplification [22]. CyHV-3 genome can also be detected and quantified in environmental water by real-time TaqMan PCR after viral concentration [85]. Recently, a mRNA-specific RT-PCR assay for detection of replicating CyHV-3 in infected fish tissues and cell cultures has been described [86]. ELISA tests have been developed to detect specific anti-CyHV-3 antibodies in carp serum [22]. CyHV-3 has been also detected in tissues and touch imprints of organs from infected fish by immunohistochemistry and immunofluorescence assays, respectively [59]. Monoclonal antibodies against CyHV-3 ORF68 have been produced. They were proved to detect specifically CyHV-3 without cross-reaction against CyHV-1 and CyHV-2 [87]. Finally, a CyHV-3-detection kit (The FASTest® Koi HV kit) that allows the detection of CyHV-3 in gill swabs in just 15 min has been developed [88].

### 3.6 Vaccination

Soon after the identification of CyHV-3 as the causative agent of koi herepsvirus disease (KHVD), an original protocol was developed to induce a protective adaptive immune response in carp [11]. This approach exploited the fact that CyHV-3 induces fatal infections only when temperature is between 18 °C and 28 °C. According to this protocol, healthy fingerlings are exposed to the virus by cohabitation with sick fish for 3–5 days at permissive temperature (22 °C-23 °C). After that the fish are transferred to ponds for 25–30 days at non-permissive water temperature (≈30 °C). Despite its ingenuity, this protocol has several disadvantages. (*i*) Fish that are “vaccinated” with this protocol become latently infected by a virulent strain and are therefore likely to represent a potential source of CyHV-3 outbreaks if they later cohabitate with naïve carp. (*ii*) The increase of water temperature to non-permissive is costly and correlated with increasing susceptibility of the fish to secondary infection. (*iii*) Finally, after this procedure only 60% of immunized fish proved to be resistant to a CyHV-3 challenge performed by cohabitation with infected fish [11].

Attenuated live vaccines appear to be the most appropriate for mass vaccination of carp. Live attenuated vaccine candidates have been produced by serial passages in cell culture of a pathogenic strain. A vaccine strain candidate was further attenuated by UV irradiation in order to increase random mutations throughout the genome [11,89]. Currently, a live attenuated vaccine developed using this approach has been manufactured by KoVax Ltd. (Jerusalem, Israel) and is available for immersion vaccination of common and koi carp in Israel [90]. Protection against CyHV-3 is associated with elevation of specific antibodies against the virus [11,89]. However, the duration of the protection conferred by the vaccine has not been established [90]. This vaccine has two major additional disadvantages: (*i*) the determinism of the attenuation is unknown; and consequently, reversions to a pathogenic phenotype cannot be excluded; (*ii*) the attenuated strain retains residual virulence that could be lethal for a portion of the vaccinated fish [91], particularly for small/young fish.

An inactivated vaccine candidate was also described by Yasumoto et al. [92]. It consists of formalin-inactivated CyHV-3 trapped within a liposomal compartment. This vaccine can be used for oral immunization in fish food. Protection efficacy for carp was 70% [92].

## 4. Host-pathogen interactions

### 4.1 Genetic resistance of carp strains to CyHV-3

Genetic differences in resistance to CyHV-3 have been described among different carp strains and crossbreeds. Independent research groups demonstrated that resistance to CyHV-3 can be significantly increased by crossing of domesticated carp strains with wild carp strains. Shapira et al. reported that the most resistant carp crossbreed in their study (60% of survival) was that between the domesticated carp strain Dor-70 and the wild carp strain Sassan [93]. In comparison the survival rate of domesticated carp strains Našice and Dor-70 as well as their crossbreed was much lower (8%, 27% and 17.7%, respectively) [93]. Recently, Piačková et al. demonstrated that most of Czech strains and crossbreeds which are genetically related to wild Amur carp were significantly more resistant to CyHV-3 infection than strains with no relation to Amur carp [94]. Carp genetic resistance to CyHV-3 has been investigated using 96 carp families derived from diallelic crossing of two wild carp strains (Amur and Duna) and two domesticated Hungarian strains (Tat and HAKI 15) [95]. This study demonstrated that crossing with wild carp strains may result in higher resistance to CyHV-3. However, individual parents of the strains are also important since many of the families derived from the wild strains did not exhibit significantly higher resistance [95]. Recently, resistance to CyHV-3 has been also linked to the polymorphism of the MHC class II *B* genes [56] and carp IL-10 gene [96]. These findings support the hypothesis that the outcome of the disease can be controlled in some extent by genetic factors of the host, and consequently, that selection of resistant carp breeders is one of potential ways to reduce the negative impact of CyHV-3 on carp aquaculture.

### 4.2 Immune response of carp against CyHV-3

Knowledge on the immune mechanisms and immunological traits that can correlate with disease resistance in fish as well as on the immune evasion mechanisms expressed by CyHV-3, is essential for the development of prophylactic strategies (such as vaccination) as well as for the development of more resistant strains by the use of molecular marker assisted selection. The information related to these topics are summarized in this section.

Perelberg et al. studied the kinetic of anti-CyHV-3 antibody expression in the serum of carp infected at different temperatures [91]. In fish that were infected and maintained at 24 °C, antibody titers began to rise at 10 dpi and reached a peak around 20-40 dpi. It was shown that protection against CyHV-3 is proportional to the titer of specific antibodies produced during the primary infection. The level of antibodies decreased in the absence of antigenic re-exposure. At 280 dpi, the titer of anti-CyHV-3 antibodies of infected fish was only slightly higher or comparable to that of unexposed fish. Nevertheless, immunized fish, even those in which antibodies were no longer detectable were resistant to a lethal challenge; possibly because of the subsequent rapid response of B and T memory cells to antigen re-stimulation [91].

Recently, a transcriptomic study uncovered the wide array of immune-related genes involved in the anti-CyHV-3 immune response of carp [97]. The response of two carp lines with different resistance to CyHV-3 has been studied using DNA microarray and real-time PCR. Significantly higher expression of several immune-related genes including number of those which are involved in pathogen recognition, complement activation, MHC class I-restricted antigen presentation and development of adaptive mucosal immunity was noted in more resistant carp line. Further real-time PCR based analyses provided evidence for higher activation of CD8^+^ T cells in the more resistant carp line. Thus, differences in resistance to CyHV-3 can be correlated with differentially expressed immune-related genes [97].

The anti-CyHV-3 immune response has been studied in the skin and the intestine of common carp [60,98]. These studies revealed an up-regulation of pro-inflamatory cytokine IL-1β, the inducible nitric oxide synthase (iNOS) and activation of interferon class I pathways [60,98]. In skin, CyHV-3 infection leads to down-regulation of genes encoding several important components of the skin-mucosal barrier, including antimicrobial peptides (beta defensing 1 and 2), mucin 5B, and tight junction proteins (claudin 23 and 30). This probably contributes to changes in the skin bacterial flora and subsequent development of secondary bacterial infections [60]. Raj et al. demonstrated that skin mucus also acts as an innate immune barrier and inhibits CyHV-3 binding to epidermal cells at least partially by neutralisation of viral infectivity [75]. In vitro study demonstrated that CyHV-3 inhibits activity of stimulated macrophages and proliferative response of lymphocytes and that this effect is temperature dependent [99].

#### 4.2.1 Interferon type I response

Interferons (IFNs) are secreted mediators that play essential roles in the innate immune response against viruses. In vitro studies demonstrated that CyHV-3 inhibits IFN type I secretion in CCB cells [100]. Poly I:C stimulation of CCB cells prior to CyHV-3 infection activated the IFN type I response and reduced CyHV-3 spreading in the cell culture [100]. In vivo studies showed that CyHV-3 induced a systemic IFN type I response in carp skin and intestine and that the magnitude of IFN type I expression is correlated with the virus load [60,98].

Recently, Tomé et al. demonstrated that CyHV-3 ORF112 encodes a new Z-domain family protein which in vitro showed structural and functional properties similar to the poxvirus E3L inhibitor of interferon response [101]. This suggested that CyHV-3 may use similar mechanisms to inhibit interferon response as poxviruses. However, the potential function of ORF112 in virus pathogenesis in vivo has not been studied yet.

#### 4.2.2 The role of CyHV-3 IL-10 homologue

CyHV-3 ORF134 encodes a viral homologue of cellular IL-10 [18]. Its expression product is a 179 amino acid protein [102]. Common carp IL-10 and CyHV-3 IL-10 exhibit 26.9% identity (67.3% similarity) over a 156 amino acid overlap [103]. Transcriptomic analyses revealed that ORF134 is expressed as a spliced gene belonging to the early [37] or early-late class [31]. Proteomic analyses of CyHV-3 infected cell supernatant demonstrated that ORF134 expression product is one of the most abundant proteins of the CyHV-3 secretome [31]. In CyHV-3 infected carp, ORF134 is highly expressed during acute and reactivation phase, while is expressed on a low level during low-temperature induced persistent phase [102]. In vivo study using a zebrafish embryo model suggested that CyHV-3 ORF134 encodes a functional IL-10 homologue [102]. Injection of mRNA encoding CyHV-3 IL-10 into zebrafish embryos increased the number of lysozyme-positive cells to a similar degree as observed with zebrafish IL-10 [102]. Moreover, down-regulation of the IL-10 receptor long chain (IL-10R1) using a specific morpholino abrogated the increase of the number of lysozyme-positive cells after co-injection with either CyHV-3 IL-10 mRNA or zebrafish IL-10 mRNA, indicating that it functions via the IL-10 receptor [102].

Recently, a CyHV-3 strain deleted for ORF134 and a derived revertant strain were produced using BAC cloning technologies [31]. The recombinant ORF134 deleted strain replicated in vitro comparably to the parental and the revertant strains. Infection of fish by immersion in water containing the virus induced comparable mortality for the three virus genotypes tested (wild type, deleted and revertant). Quantification of viral DNA by real time TaqMan PCR and analysis of carp cytokines expression by RT-qPCR at different times post-infection did not reveal any significant difference between the groups of fish infected with the three virus genotypes. Moreover, histological examination of infected fish did not reveal significant differences between fish infected with the three genotypes. Altogether, these results demonstrated that the IL-10 homologue encoded by CyHV-3 is essential neither for viral replication in vitro nor for virulence in vivo [31].

## 5. Conclusions

Since its first description in the late 1990s, CyHV-3 rapidly spread to different continents (Europa, Asia, North America, Africa) causing severe financial losses in the common carp and koi culture industries worldwide. In addition to its negative economical and societal impacts, CyHV-3 has also a negative environmental impact by affecting wild populations of carp. These reasons explain why CyHV-3 became rapidly a subject for applied science and is now listed as a notifiable disease by the OIE. In addition to its economic importance, recent studies demonstrated that CyHV-3 is also a very attractive and original subject of fundamental research: (*i*) it is phylogenetically distant from the vast majority of herpesviruses that have been studied so far (the latter belong to the family *Herpesviridae*), thereby providing an original field of research. (*ii*) It can be studied in laboratories by infection of its natural host (homologous virus-host model). (*iii*) The sequence of its genome published recently revealed a fascinating virus with unique properties in the *Herpesvirales*, such as an extremely large genome (295 Kb), a high number of genes which are not homologous to known viral sequences, and genes that are normally found exclusively in the *Poxviridae*[18]. (*iv*) Importantly, the CyHV-3 genome revealed several genes encoding proteins potentially involved in immune evasion mechanisms. (v) Last but not least, the outcome of CyHV-3 infection is highly dependent on the temperature of the water in which the carp are maintained.

## 6. Abbreviations

AngHV-1: Anguillid herpesvirus 1; Au: Goldfish fin cell; BAC: Bacterial artificial chromosome; CaF-2: Carp fin cell; CCB: *Cyprinus carpio* brain cell; CCG: *Cyprinus carpio* gill cell; CNGV: Carp interstitial nephritis and gill necrosis virus; CPE: Cytopathic effect; CyHV-1: Cyprinid herpesvirus 1; CyHV-2: Cyprinid herpesvirus 2; CyHV-3: Cyprinid herpesvirus 3; dUTPase: Deoxyuridine triphosphate pyrophosphatase; FHM: Fathead minnow cell; iNOS: Inducible nitric oxide synthase; IFNs: Interferons; IL-1β: Interleukin 1β; IL-10: Interleukin 10; KFC: Koi fin cell; KF-1: Koi fin cell; KHV: Koi herpesvirus; KHVD: Koi herpesvirus disease; LUC: Luciferase; MHC class II B: Major histocompatibility complex class II *B*; NGF-2 and NGF-3: Epithelial-like cell line from fins of coloured carp (2 and 3); ORF: Open reading frame; TK: Thymidine kinase; TNFR: Tumor necrosis factor receptor; Tol/FL: Silver carp fin cell; VNTR: Variable number of tandem repeats; 2D-LC MS/MS: Two-dimensional liquid chromatography tandem mass spectrometry.

## 7. Competing interests

Dr Vanderplasschen’s group is developing vaccine candidates against CyHV-3 as well as reagents to diagnose the disease.

## 8. Authors’ contributions

KR, PO, and AV contributed to the design of the structure of the manuscript. KR and AV drafted the manuscript. KR, PO, MB, MR, AR, CV, JJ-R, and AV performed the overview of the literature on CyHV-3, read and approved the final manuscript.
